# Vascular contribution to cognitive impairment and dementia (VCID): proceedings of 2025 workshop of the Jackson Laboratory

**DOI:** 10.1007/s00335-026-10210-x

**Published:** 2026-03-14

**Authors:** Mehwish Anwer, Tetiana Poliakova, Ricardo D’Oliveira Albanus, Mohammad Iqbal H. Bhuiyan, Adam M. Brickman, Soumilee Chaudhuri, Leah Cuddy, Maxwell Eisenbaum, Kate Foley, Douglas B. Gould, Catherine Hall, Costantino Iadecola, Olivia Marola, Christopher M. Norris, Alaina Reagan, Julie Schneider, Donna Wilcock, Isabella Xu, Andrew Yang, Gareth Howell, Cheryl L. Wellington

**Affiliations:** 1 Department of Pathology and Laboratory Medicine, University of British Columbia, Vancouver, Canada; 2 Djavad Mowafaghian Centre for Brain Health, University of British Columbia, Vancouver, Canada; 3 The Jackson Laboratory, Bar Harbor, ME USA; 4 International Collaboration on Repair Discoveries, University of British Columbia, Vancouver, Canada; 5 School of Biomedical Engineering, University of British Columbia, Vancouver, Canada; 6 Department of Neurology, Washington University School of Medicine, St. Louis, USA; 7 Department of Neurology, LSU Health, Shreveport, Louisiana USA; 8 Taub Institute for Research on Alzheimer’s Disease and the Aging Brain, Department of Neurology, Vagelos College of Physicians and Surgeons, Columbia University, New York, USA; 9 Indiana University, Bloomington, USA; 10 Ken and Ruth Davee Department of Neurology, Northwestern University Feinberg School of Medicine, Chicago, IL 60611 USA; 11 The Roskamp Institute, Sarasota, FL USA; 12 Indiana University School of Medicine, Indianapolis, USA; 13 Department of Ophthalmology, Department of Anatomy, Cardiovascular Research Institute, Bakar Aging Research Institute, and Institute for Human Genetics, University of California, San Francisco, USA; 14 School of Psychology, University of Sussex, Brighton and Hove, UK; 15 British Heart Foundation - UK Dementia Research Institute Centre for Vascular Dementia Research, University College London, London, UK; 16 Feil Family Brain and Mind Research Institute, Weill Cornell Medicine, New York, USA; 17 University of Kentucky College of Medicine, Lexington, USA; 18 Department of Pathology and Laboratory Medicine Djavad Mowafaghian Centre for Brain Health, University of British Columbia, Vancouver, Canada; 19 Rush University, Chicago, USA; 20 Washington University, St. Louis, USA; 21 Gladstone Institutes/UCSF, San Francisco, USA

**Keywords:** Vascular contributions to cognitive impairment and dementia (VCID), Alzheimer’s disease and related dementias, Neurovascular pathology, Interdisciplinary collaboration, Career development, Trainee workshop

## Abstract

The first Vascular Contributions to Cognitive Impairment and Dementia (VCID) Workshop, held on May12-16, 2025 at The Jackson Laboratory, provided scientific content and career development training in the latest developments in VCID and Alzheimer’s Disease (AD) and related dementia (ADRD). The Workshop aimed to foster interdisciplinary collaborations and empower the next generation of diverse scientists to advance ADRD research from mechanism to intervention. The meeting placed a strong emphasis on neurovascular pathology, its central role in ADRD pathogenesis, its modulation by both central and peripheral factors, and the critical need to dissect the mechanistic basis of VCID through experimental models. The workshop included talks from both faculty and trainees and was bookended by two keynote addresses by Drs. Donna Wilcock and Costantino Iadecola, leaders and pioneers in the VCID field. The second VCID workshop is planned for April 28-May 1, 2026, to be held at Washington University in St. Louis.

## Introduction

Vascular disease is extraordinarily common in the aging brain and emerging evidence supports vascular impairments as major contributors to cognitive decline and dementia, including Alzheimer’s disease (AD) and related dementias (ADRDs). Vascular dementia is commonly deemed the second most common dementia subtype, representing 15–20% of cases, where cerebrovascular disease may contribute to cognitive and functional decline in as many as 75% of individuals with dementia (Sachdev et al. 2025). Despite its prevalence, progress in research and clinical practice has remained limited.

Hosted by The Jackson Laboratory in Bar Harbor, Maine with funding from the Diana Davis Spencer Foundation and the National Institute of Neurological Disorders and Stroke, the 2025 Vascular Contributions to Cognitive Impairment and Dementia (VCID) Workshop convened experts from multiple disciplines to discuss challenges in VCID pathophysiology and treatment. The workshop brought together scientists across the career spectrum, including graduate students, postdoctoral fellows, and junior and senior faculty, from the fields of vascular biology, neurodegeneration, genetics of ADRD, and computational biology. The meeting had a particular focus on neurovascular pathology, including the crucial role it plays in AD and ADRD pathogenesis and how it can be affected by central and peripheral influences. A critical need for understanding the mechanistic underpinnings of VCID for development of robust biomarkers and interventions was identified.

### Trainee-centered learning

A central objective of the VCID Workshop was to promote meaningful interactions that foster collaborative research and support career advancement among early-career investigators. To achieve this goal, the program emphasized active, trainee-centered learning and engagement through multiple complementary formats, including trainee presentations, a grant proposal exercise, structured feedback sessions, and professional skill development workshops.

Trainee short talks were integrated throughout the program along with a poster session providing participants with multiple opportunities to present their research to an interdisciplinary audience of peers and senior investigators. A cornerstone of the workshop was the structured grant proposal development exercise, designed to strengthen trainees’ skills in conceptualizing, refining, and communicating research ideas. Participants worked in groups on proposal aims with guidance from workshop faculty, where they received targeted feedback on scientific rigor, feasibility, and alignment with funding priorities. Professional development was further supported through an interactive workshop on scientific communication and elevator pitches, led by Bri McWhorter, founder and CEO of *Activate to Captivate LLC*. This session equipped trainees with practical strategies for delivering clear, compelling research narratives to diverse audiences, an essential skill for career advancement in science related fields. Collectively, these active training and engagement activities created a highly interactive environment that encouraged collaboration, mentorship, and professional growth. By integrating knowledge exchange with hands-on skill development, the workshop effectively advanced its goal of fostering a collaborative VCID research community while supporting the career trajectories of early-career investigators.

## Intersections of vascular pathology and Alzheimer’s disease

Dr. Donna Wilcock, PhD (Indiana University) delivered the opening keynote address and introduced the audience to the greater VCID field. Dr. Wilcock reviewed that vascular contributions to dementia include cerebral small vessel disease (cSVD), cerebral amyloid angiopathy (CAA) and blood-brain barrier (BBB) dysfunction. Traditionally, vascular episodes leading to dementia have been characterized as vascular dementia, with AD characterized as neurodegeneration in the face of amyloid plaques and neurofibrillary tangles (NFTs). However, multiple studies are now showing that ADRDs are more of a spectrum of disorders and include mixed etiology dementias where multiple pathologies are present in the diseased brain (Sachdev et al. 2025). To elucidate the causal changes, the fields of vascular health and ADRD are taking similar strategies that include genetic and biomarker studies in humans and model organisms, and research is converging with the use of recent advances in genomic technologies (e.g., single-cell RNA-seq and proteomics) that allow more precise description of molecular and cellular changes that occur as a function of age, cardiovascular and cerebrovascular health, neuroinflammation, and neurodegeneration.

Dr. Julie Schneider, MD, MS (Rush University) discussed that large community-based neuropathology cohorts including the Religious Orders Study and the Rush Memory and Aging Project (ROSMAP), as well as the Minority Aging Research Study (MARS) provide unparalleled longitudinal clinical data and brain autopsy resources for understanding dementia and VCID (Bennett et al. 2018; Schneider and Bennett 2010). These studies, which follow participants prospectively, have generated one of the largest collection of autopsies. Across these cohorts, common age-related pathologies include amyloid plaques, Lewy bodies, and widespread vascular lesions; importantly, many infarcts are microscopic, escaping detection by neuroimaging or gross examination (Arvanitakis et al. 2011). Dr. Schneider’s work highlights that watershed regions, which are highly susceptible to hypoxic-ischemic stress are often overlooked in AD research, harbor a substantial burden of infarcts (Kapasi et al. 2018). She noted that atherosclerosis and arteriosclerosis of the Circle of Willis vessels are frequent in older adults, underscoring the pervasive role of vascular disease in late-life cognitive decline (Arvanitakis et al. 2016). Shared risk pathways further link chronic traumatic encephalopathy (CTE), traumatic brain injury (TBI), and diet to vascular brain injury, while CAA, although distinct from AD, shares genetic risk factors such as the e4 allele of the apolipoprotein E gene (*APOE4*), reinforcing the intertwined nature of vascular and neurodegenerative mechanisms (Corriveau et al. 2016).

While discussing the complexity of vascular dysfunction, Dr. Costantino Iadecola, MD (Weill Cornell Medicine) who gave the closing keynote presentation (see below), also highlighted in his address that cerebral blood vessels are not normal in AD and that cerebral blood flow (CBF) is reduced early in the course of the disease (Iadecola 2004). Evidence from the Nun study illustrates that vascular injury in the form of lacunar and larger brain infarcts aggravate dementia in patients with mild AD pathology (Snowdon et al. 1997). Dr. Iadecola stressed that there is ample evidence of an interaction between vascular cognitive impairment and AD. For example, proteomic sequencing of the dorsolateral prefrontal cortex in 438 older individuals revealed that cerebral atherosclerosis has a detrimental effect on cognition independent of amyloid-beta (Aβ) and via mechanisms shared with tau (A. P. Wingo et al. 2020).

### White matter hyperintensities and vascular damage

Growing evidence underscores a direct and multifaceted role of cerebrovascular disease in AD, extending beyond comorbidity to an integral part of disease pathogenesis and progression. Dr. Adam Brickman, PhD (Columbia University) highlighted that vascular injury contributes additively, synergistically, and directly to AD pathogenesis, likely via tau, and clinical course in late-onset and populations with genetically deterministic forms of AD. The vascular supply through the brain is not uniform, and white matter is particularly vulnerable due to its reliance on small, distal arterioles that are highly sensitive to vascular compromise. Findings from several observational studies demonstrated that greater white matter hyperintensity (WMH) burden, especially in posterior regions, increased the risk of incident AD or dementia, and longitudinal investigation of WMH revealed increased risk of conversion (Brickman et al. 2012, 2015).

In amyloid-positive individuals, only those with elevated WMH showed symptomatic manifestations of AD (Provenzano et al. 2013), and higher parietal and occipital WMH volumes were associated with accelerated cerebrospinal fluid (CSF) tau accumulation (Tosto et al. 2015). Imaging of older adults in the Washington Heights Inwood Columbia Aging Project revealed that those with parental, particularly maternal, history of dementia have increased WMH, further exacerbated in the *APOE4* carriers, reinforcing their link to genetic vulnerability (Stamm et al. 2020).

Evidence suggests that vascular pathology emerges early in autosomal dominant AD prior to expected symptom onset (Lee et al. 2016) and in Down syndrome-associated AD, often preceding or coinciding with amyloid and tau deposition (Lao et al. 2020; Natalie C. Edwards et al. 2025b). Experimental studies in mice support this temporal sequence: transient middle cerebral artery occlusion in aged mice induced increased plasma and CSF tau concentrations, induced myelin loss, and hyperphosphorylated tau pathology in the ipsilateral hippocampus and cerebral hemisphere, further implicating vascular stress as a driver of tau pathology (Laing et al. 2020). These studies support cerebrovascular disease as a core feature of AD and not simply a comorbidity. An ongoing study by the Brickman team is investigating the relationship between vascular injury, white matter damage and inflammation using translocator protein positron emission tomography (TSPO PET) to reveal microglial activation.

Previous studies using emerging pathway analysis connect WMH to sequential molecular changes, specifically, rising glial fibrillary acidic protein (GFAP) as an astrocytic response, followed by phosphorylated tau (p-tau) accumulation and later neurofilament light (NfL) elevation as a marker of neurodegeneration (N. C. Edwards et al. 2025c). Placental growth factor (PlGF) appears to statistically drive both WMH and GFAP, implicating inflammation and blood-brain barrier (BBB) disruption, rather than traditional peripheral vascular risks, as upstream contributors (Natalie C. Edwards et al. 2025a).

Collectively, these findings highlight that cerebrovascular disease constitutes a fundamental feature of AD rather than a secondary comorbidity. The recognition of an endogenous vascular component independent of hypertension, diabetes, or other systemic risks shifts attention toward intrinsic neurovascular unit dysfunction and BBB integrity as key therapeutic targets. Integrating neuroimaging and fluid biomarkers provides a powerful approach to disentangle these mechanisms, refine risk stratification, and inform vascular-targeted strategies for AD treatment and prevention.

### CAA

Dr. Olivia Marola, PhD, a postdoctoral associate in Dr. Gareth Howell’s lab (The Jackson Laboratory) presented data exploring how genetically diverse mouse strains shape susceptibility to CAA, a pathology present in 90% of AD patients (Grinberg and Thal 2010). Traditionally used dementia models on a C57BL/6J (B6) genetic background show limited susceptibility to CAA, and the Howell Lab previously demonstrated that wild-derived WSB/EiJ (WSB) mice develop age-dependent, human-relevant CAA in the presence of the *APP/PS1* amyloid driver (Onos et al. 2019, 2025). Specifically, WSB.*APP/PS1* mice show mild CAA accompanied by blood flow and metabolic deficits, and modifications in small vessel morphology as seen by PET/CT at 8 months of age progressing to robust CAA at 14 months (Onos et al. 2025). This model exhibits low parenchymal plaque burden but robust vascular amyloid deposition, contrasting the typical phenotype seen in B6.*APP/PS1* mice.

To dissect the genetic contributions to CAA, Dr. Marola crossed WSB mice with B6.*APP/PS1* to generate F1 BXW.*APP/PS1* offspring, which exhibit more advanced CAA compared to founder strains at 8 months, which illustrates that heterozygous loci facilitate the development of CAA. BXW.*APP/PS1* mice also demonstrate high parenchymal plaques, high plasma amyloid and no drastic neuronal loss. Intercrossing F1 mice produced genetically diverse F2 offspring that exhibited variable severity of CAA, which suggests CAA-susceptibility is likely identifiable through genetic mapping. An initial attempt to protect the BBB and mitigate CAA by reducing Angiopoietin-2 (ANGPT2) expression through heterozygosity did not attenuate vascular pathology in these mice, suggesting ANGPT2 deficiency alone is insufficient to have a therapeutic effect in this context. Together, these findings establish WSB.*APP/PS1* mice as a robust platform to investigate the genetic and molecular mechanisms underlying CAA and AD-related vascular deficits and emphasize the importance of incorporating genetic diversity into preclinical models of AD.

Amyloid-beta deposition is a pathological hallmark of AD and has been studied extensively for its contribution to disease outcome. However, the processes underlying Aβ cellular processing and deposition need further investigation. Isabella Xu, MD-PhD student in Dr. Jin-Moo Lee’s laboratory at University of Washington, introduced a novel fluorescence-based strategy to visualize where Aβ is produced within cells, addressing a long-standing limitation in AD research. Aβ is generated by sequential β- and γ-secretase cleavage of amyloid precursor protein (APP), yet the intracellular sites of this processing remain unclear due to the difficulty of distinguishing newly cleaved Aβ from full-length APP. By combining unnatural amino acid mutagenesis with click chemistry to selectively label Aβ within an engineered APP construct, super-resolution imaging and object-based quantification dynamic separation of APP and Aβ signals during trafficking were revealed (Xu et al. 2023). Overall, this platform provides a qualitative and semi-quantitative map of Aβ localization by resolving the spatial divergence of APP and its cleavage products inside cells. Coupling this approach with subcellular markers will refine localization within the endolysosomal system and may be extended to primary neurons to study Aβ trafficking in a physiologically relevant architecture as well as vascular and perivascular cells to study mechanisms underlying CAA and impaired perivascular clearance, key vascular components of cognitive impairment and dementia.

## Neurovascular unit and VCID pathology

The neurovascular unit (NVU) comprising endothelial cells, pericytes, astrocytes, vascular smooth muscle cells, neurons, and immune cells play a central role in maintaining CBF, blood brain barrier (BBB) integrity, and metabolic homeostasis. In VCID, dysfunction of the NVU is increasingly recognized as a unifying mechanism linking vascular injury to progressive cognitive decline (Lin et al. 2025). Pathological processes such as hypertension, chronic hypoperfusion, small vessel disease, and inflammatory signaling disrupt endothelial-pericyte interactions, alter tight junction composition, and impair autoregulatory control of blood flow, leading to BBB leakage, white matter damage, and neuronal stress (Wardlaw et al. 2017; Procter et al. 2021). Together, these NVU-driven alterations create a feed forward cascade of neurovascular dysfunction that underlies the clinical and structural heterogeneity of VCID, positioning the NVU as a critical therapeutic target for slowing or preventing vascular cognitive decline.

Dr. Costantino Iadecola, MD (Weill Cornell University) started his keynote address highlighting the many roles of the highly-regulated neurovasculome including temporal- and spatial-specific coupling of neural activity to blood flow, endothelial regulation and BBB function, waste clearance to the peripheral vasculature (e.g. Aβ and tau), immune homeostasis and trophic support (Monica M. Santisteban and Iadecola 2025). Because of this variety, neurovascular dysfunction does not simply indicate the loss of cerebral blood flow; it also includes immune, clearance, and trophic dysfunctions. In AD, Aβ and tau, in addition to disrupting synaptic activity, may also contribute to the vascular dysfunction that then further amplifies the synaptic disruption and, thus, cognitive impairment.

### Border-associated macrophages

Early work from Dr. Iadecola showed that 3-month old transgenic mice overexpressing APP (Tg2576) exhibit a profound and selective deficits in endothelium-dependent regulation of the neocortical microvascular function (Costantino Iadecola et al. 1999; Niwa et al. 2000). Based on this work, Iadecola and colleagues hypothesize that, considering the sensitivity of vessels to Aβ, vascular dysfunction might be one of the earliest manifestations of AD (Iadecola 2004), which has since been confirmed in the clinical studies (Iturria-Medina et al. 2016). Mechanistic studies revealed that innate immune receptors including CD36, O_2_ radicals and transient receptor potential melastatin-2 (TRPM2) channels mediate amyloid-β-induced neurovascular dysfunction (Park et al. 2013, 2014). Notably, in Tg2576 mice, selective depletion of border-associated macrophages (BAMs) through intracerebroventricular clodronate injection abolishes reactive oxygen species generation and cerebrovascular dysfunction induced by amyloid-β – whether applied directly to the cortex, administered intravascularly, or produced endogenously in the brains of these mice (Park et al. 2017). This confirms that the main source of reactive oxygen species are the border-associated macrophages (BAM) that surround arterioles and venules. Bone marrow chimeras demonstrated that depletion of CD36 or NOX2 in BAM prevents oxidative stress and rescues neurovascular dysfunction in 3-month-old Tg2576 mice (Park et al. 2017). Even when aged to 15 months, CD36 depletion in BAM rescues neurovascular and cognitive dysfunction in these mice which was accompanied in CAA but not parenchymal plaque reduction (Uekawa et al. 2023). The same study also demonstrated that the lack of CD36 in Tg2576 mice enhanced the vascular clearance of exogenous Aβ injected into the neocortex or the striatum. Collectively, this suggests that CD36 in BAM mediate neurovascular dysfunction and promote CAA and cognitive dysfunction in APP-expressing mice.

Interestingly, BAMs express similar levels of apoE4, the greatest genetic risk factor of AD, as astrocytes, and have more apoE4 than endothelial cells, mural cells, microglia (Anfray et al. 2024). Selective *APOE4* depletion from BAM negates neurovascular dysfunction reported in mice expressing human *APOE4* (Anfray et al. 2024). *APOE4*, in addition to increasing AD risk, is also a risk factor for amyloid-induced imaging abnormalities (ARIA), a rare but potentially lethal side-effects of anti-amyloid antibody treatments for AD (Sperling et al. 2011). Considering the role of BAM in amyloid-β-induced and apoE4-medicated neurovascular dysfunction, BAMs are a possible therapeutic target in both AD and preventing ARIA.

### Astrocytes

Astrocytes play a critical role in homeostatic maintenance of brain function and are key components of the neurovascular unit and tripartite synapse in addition to acting as metabolic shuttles (Valles et al. 2023). They also play a crucial role in neuroinflammatory response to disease by acquiring a reactive morphological state with extensive ramification and through increased production of glial fibrillary acidic protein (GFAP) in brain and blood. Reactive astrocytes are also found around amyloid-beta plaques and microinfarcts indicating their role in pathogenic processes underlying AD/ADRD. Dr. Christopher M Norris, PhD (University of Kentucky) and his team are investigating how astrocytes interact with cerebral vessels to understand their role in AD/ADRD and dementias.

Previously, the Norris lab showed that inhibition of the calcium dependent calcineurin/NFAT pathway, selectively in astrocytes, resulted in reduced glial reactivity, improved synapse function, and blood flow in AD/ADRD models (Sompol et al. 2023; Furman et al. 2012). To assess astrocyte-cerebral blood vessel interactions more directly, the Norris lab utilised two photon imaging on anesthetized double transgenic APP/PS1 models of AD-like pathology and identified spontaneously hyperactive astrocytes and/or dysregulation of astrocyte calcium signaling linked to cerebrovascular dysfunction (Kuchibhotla et al. 2009; Delekate et al. 2014). However, anesthesia can profoundly affect both vascular dilation and cellular activity, which could alter the complex and highly dynamic interactions between astrocytes and cerebral vessels and obfuscate true changes associated with AD pathology (Sompol et al. 2023). To that extent, Dr. Norris argued that it is crucial to study calcium signaling of perivascular astrocytes in fully awake AD/ADRD mice. Recent work from his laboratory, using two photon imaging under fully awake conditions, confirmed that spontaneous astrocytic calcium activity is elevated in AD mice (i.e. 5xFAD) (Weiss et al. 2025). However, stimulus-evoked arteriole dilations and coordinated astrocyte end feet calcium responses are diminished compared to wild-type controls. Moreover, astrocyte network synchrony and end feet arteriole coupling is disrupted, showing slower, weaker, and less proportional calcium transients, indicating a remarkable communication breakdown between the brain and the vasculature in the context of AD pathology.

Building on the importance of astrocytic dysfunction in AD/ADRD, recent genetic studies further investigate non-neuronal pathways that may influence disease susceptibility. While investigating seven genome-wide association studies (GWAS) for AD in East Asians and the cell type–specific expression patterns using the Seattle Alzheimer’s Disease Brain Cell Atlas (SEA‐AD) dataset, Soumilee Chaudhuri, a graduate student in Drs. Kwangsik Nho and Andrew Saykin’s laboratory (Indiana University School of Medicine) identified chloride intracellular channel 4 (*CLIC4*) and potassium inwardly rectifying channel subfamily J member 6 (*KCNJ6*) as novel AD‐associated functional genes, advancing understanding of AD-related genetic risk in East Asian cohorts. Notably, expression levels of *CLIC4* were elevated in non-neuronal cell types, particularly in endothelial cells. Additionally, in astrocytes, *CLIC4* was differentially expressed according to the final diagnosis of AD (Cho et al. 2025).

These findings suggest that in AD, reactive astrocytes exhibit impaired communication with cerebral arterioles, potentially contributing to cerebral hypometabolism, neurovascular dysfunction and potentially impaired amyloid-beta clearance seen in neurodegeneration.

### Omics approaches to study different cell types

Typical RNA seq analysis can limit detection of cerebrovascular cells in unsorted single cell libraries, which necessitates data integration from multiple single-cell datasets. Therefore, development of a multi-omic single cell atlas of cerebrovascular cells can help identify neurovascular regulatory programs associated with late-onset and early-onset AD progression, identify molecular features linked to deleterious neurovascular phenotypes (e.g. CAA) and determine the contribution of vascular cells to AD genetic risk.

Dr. Andrew Yang, PhD (University of California, San Francisco) developed a new multi-omic approach, MultiVINE-seq, which enables simultaneous profiling of RNA and chromatin accessibility across vascular, perivascular, and immune cells in the human brain (Reid et al. 2025). Integrating these data with GWAS results mapped thousands of neurological disease risk variants to specific cell types and target genes, including many previously unresolved associations. The analyses reveal distinct pathogenic mechanisms: cerebrovascular disease variants primarily disrupt extracellular matrix and structural integrity pathways in endothelial, mural, and fibroblast cells, whereas AD variants alter inflammatory signalling in endothelial and immune populations. Notably, an AD risk variant increases PTK2B expression in CD8⁺ T cells, implicating adaptive immunity in AD (Reid et al. 2025). This atlas provides a foundational resource for understanding how vascular-cell genetic variation contributes to divergent neurological disease pathways.

Dr. Ricardo D’Oliveira Albanus, PhD, from Dr. Oscar Harari’s laboratory (Washington University School of Medicine) presented his ongoing investigation on molecular changes underlying AD and neurodegeneration from postmortem human tissue with a special focus on the contribution of vascular cells in AD genetic risk. Dr. Albanus and colleagues are integrating single nucleus RNA seq data from the Australian Brain Bank Network (ABBN), ROSMAP, VINE-seq, UCI MIND and others, to dissect contributions of several cerebrovascular cell populations to AD within each of these studies. These observations enable the identification of altered genes in individual cell types from AD samples, including different AD presentations (late-onset AD and autosomal-dominant AD) compared with controls. Furthermore, it was observed that *APOE4* associates with broad transcriptional changes in all vascular cell types. *APOE4* associates with vascular inflammation markers and impaired energy metabolism in endothelial cells and elicits transcriptional changes that are detectable before disease onset (D’Oliveira Albanus et al. 2024).

### Neurovascular coupling and oxygenation

The brain’s oxygen and nutrient delivery is finely tuned across the cerebrovascular network to match metabolic demand with supply. This coupling is essential for maintaining neuronal function, and growing evidence links its disruption to AD. Many established AD risk factors are associated with reductions in cerebral metabolic support, and alterations in CBF have been observed decades before the onset of clinical symptoms (Wolters et al. 2017; Iturria-Medina et al. 2016). These observations suggest that an imbalance between neuronal energy demand and vascular supply may contribute to the initiation or progression of AD pathology. It is therefore imperative to understand regional vulnerability to vascular dysfunction, its impact on neuronal activity and integrity, and its contribution to development of AD and related dementias. The hippocampus, a region critical for spatial and episodic memory, is highly vulnerable to hypoxia and is affected early in AD. Dr. Catherine Hall, PhD (University of Sussex, UK-DRI at University College London) utilized in vivo 2-photon imaging to identify that, compared with neocortex, the hippocampus exhibits lower blood flow, reduced oxygenation, and weaker neurovascular coupling, driven by differences in vascular architecture and pericyte/endothelial function (Shaw et al. 2021). Oxygen-diffusion modelling suggested that these vascular features may limit metabolic supply, providing a mechanistic basis for the heightened sensitivity in the hippocampus to injury and neurodegenerative processes (Shaw et al. 2021).

Studies using *APOE*-targeted replacement mice showed that *APOE4* carriers exhibit impaired neurovascular responses, with blunted activity-evoked increases in blood flow, volume, and oxygenation due to frequent vessel dilation failures. These deficits created a mismatch between metabolic demand and vascular supply. Dr. Hall further investigated if *APOE4* genotype and lifestyle interact to drive neurovascular dysfunction. Physical activity improved neurovascular function in a dose-dependent manner, with particularly pronounced benefits in *APOE4* mice. Together, these findings suggested that the combination of *APOE4* genotype and sedentary lifestyle produces the most severe cerebrovascular dysfunction, highlighting physical activity as a potentially powerful modifier of dementia risk in *APOE4* carriers (Anderle et al. 2025).

Dr. Leah Cuddy, PhD (Northwestern University) presented new evidence implicating the neuronal renin–angiotensin system (RAS) in neurovascular dysfunction and AD. Expressed at significant levels in all human tissues including the brain, angiotensin I converting enzyme (ACE1) converts angiotensin I into angiotensin II (AngII) in the RAS maintaining the blood pressure homeostasis (Takimoto-Ohnishi and Murakami 2019). In addition to its established role in the cardiovascular system, growing evidence suggests a distinct neuronal function that may contribute to AD pathology and neurodegeneration (Kamath et al. 2022; Savaskan et al. 2001; Villar-Cheda et al. 2010). Multiple large scale genomic and proteomic association studies highlighted ACE1 as a significant genetic contributor to AD risk (Ge et al. 2023; Jansen et al. 2019; Kunkle et al. 2019). Dr. Cuddy’s presentation focused on the ACE variant rs4980 (R1279Q human and R1284Q murine mutation) discovered in LOAD families through whole genome sequencing. In knock-in mice (KI), ACE1 R1284Q increases brain ACE1 and AngII, but blood pressure remains normal, suggesting a brain-specific mechanism of action (Cuddy et al. 2020). These mice developed hippocampal neurodegeneration and memory impairment with more profound effects in female mice that were prevented by ACE inhibitors (captopril) and angiotensin receptor blockers (losartan), suggesting therapeutic relevance of targeting neuronal ACE1 signaling. Young ACE1 R1284Q mice also demonstrated increased fibrinogen deposition while aged mice showed vascular abnormalities including increased astrocyte-vessel distance (Cuddy et al. 2020).

Using neuron-specific conditional knockouts of ACE1, Cuddy and colleagues demonstrated that excitatory neurons are the predominant source of ACE1 expression and activity in the hippocampus, which suggests that excitatory neurons are the predominant contributors to ACE1-mediated AngII production in the hippocampus (Jeon et al. 2024). ACE1 conditional knock-out mice exhibit hippocampus-dependent memory deficits and cerebrovascular loss with age in hippocampus (Jeon et al. 2024). Cuddy and the group also investigated loss of neuronal angiotensin II type 1 receptor (AT1R) in mice and increased fibrinogen deposition. In summary, Dr. Cuddy highlighted that astrocytic coverage deficits and vascular tortuosity point to dysregulated neuron-to-vessel signaling, possibly implicating ACE1-AngII-AT1R in NVU coordination and leading to neurodegeneration in ACE1 R1284Q mice.

### Neurotrauma and VCID

Dr. Mehwish Anwer, PhD, a postdoctoral fellow in Dr. Cheryl Wellington’s laboratory at The University of British Columbia discussed that traumatic brain injury (TBI) results in blood brain barrier damage and CBF impairments, which can lead to cognitive impairment and dementia including ADRDs. Therefore, a detailed analysis of the molecular and cellular changes that occur as a function of injury, age, cerebrovascular health, neuroinflammation, and neurodegeneration is crucial to understanding of vascular contributions to dementia. Dr. Anwer emphasised that high throughput approaches like spatial transcriptomics can provide detailed molecular maps of spatial gene dysregulation and shared recent findings on cell type specific gene dysregulation in white matter tracts of mice with mild TBI. Region specific upregulation of the astrocytic marker *Gfap*, microglial marker *Aif1* and several disease-associated microglia (DAMs) genes including *Apoe*, *Ctsd*, *Trem2* was observed (Swaro et al. 2025; Anwer et al. 2025), generating novel avenues into research on TBI-induced VCID.

Recent evidence underscores the pivotal role of cerebrovascular mural cells, particularly pericytes and smooth muscle cells, in maintaining neurovascular integrity and mitigating secondary pathologies following TBI. Closed head injury mouse models of repetitive mild TBI, including CHIMERA (Closed Head Impact Model of Engineered Rotational Acceleration), have shown chronic gliosis, myelin loss, axonal damage, tau accumulation, impaired cerebral blood flow and impaired spatial learning and memory (Abrahamson and Ikonomovic 2020; Ramos-Cejudo et al. 2018; Mouzon et al. 2018; Cheng et al. 2019; Bashir et al. 2020). However, little is known about mechanisms underlying vascular damage and interaction of tau with BBB. Dr. Maxwell Eisenbaum, PhD, a postdoctoral fellow with Dr. Fiona Crawford at the Roskamp Institute, presented his work on role of endothelial dysfunction in chronic outcomes of mild TBI with particular focus on interaction of tau with cerebrovasculature and VCID modifying risk factors like age and *APOE4*.

Dr. Eisenbaum showed that endothelial transcytosis contributes to the removal of pathogenic tau from the brain and identified multiple endothelial transporters capable of facilitating tau transit, including caveolin-1 (Eisenbaum et al. 2021) and the apoE receptor, LRP1 (Eisenbaum et al. 2024). Repeated exposure to mTBI resulted in chronic endothelial cell dysfunction, characterized by reduced endothelial caveolin-1 expression, impaired caveolar-mediated cerebrovascular tau uptake (Ojo et al. 2021), and attenuated vascular clearance of exogenous tau from the brain following intracortical injection (Eisenbaum et al. 2021).

This work positions endothelial cell dysfunction as a central mechanism linking TBI to tauopathies and highlights it as a potential therapeutic target. Complementary research using *APOE*-targeted replacement mice revealed that vascular tau clearance from the brain was diminished in *APOE4* mice, which could be attributed to the inhibitory impact of apoE4 on LRP1-mediated tau uptake (Eisenbaum et al. 2024). In addition to the deficits in tau transit, repetitive mTBI precipitated chronic eNOS dysregulation, mural cell degeneration, gliovascular dissociation, and glymphatic suppression (Eisenbaum et al. 2024). Collectively, these studies advance the understanding of vascular contributions to TBI pathology and may therefore hold promise in mitigating chronic neurovascular and neurodegenerative sequelae of brain trauma.

White matter lesions (WML), BBB disruptions and astrogliosis are major drivers of VCID. The molecular pathways that link chronic hypoperfusion to these pathologies remain unclear. Activation of the Na-K-Cl cotransporter 1 (NKCC1) by its upstream kinase SPAK (STE20/SPS1-related proline/alanine-rich kinase) promotes intracellular sodium overload, astrocyte hypertrophy, and gliosis, suggesting a potential mechanism in VCID. Dr. Bhuiyan (LSU Health, Shreveport) tested whether the SPAK inhibitor ZT-1a could mitigate established pathology using a bilateral carotid artery stenosis mouse model (Habib et al. 2025). Treatment during the symptomatic phase improved cerebral blood flow and rescued learning and memory deficits. ZT-1a reduced astrogliosis, microglial activation, MMP2 and MMP9 expression, BBB breakdown, demyelination and neurodegeneration (Habib et al. 2025). These findings indicate that hypoperfusion-induced SPAK-NKCC1 signaling drives gliovascular dysfunction and cognitive decline, identifying this pathway as a promising therapeutic target for VCID.

## Peripheral and systemic influences

Peripheral and systemic factors emerged as important modulators of VCID. Dr. Cheryl Wellington, PhD (University of British Columbia, and co-organizer of the workshop) highlighted the interplay between cerebrovascular pathologies, cardiometabolic risk factors, lifestyle influences, and social determinants of health in shaping dementia trajectories, emphasizing opportunities for intervention.

### Lipoproteins

*APOE4* remains the strongest genetic risk factor of late-onset AD, with both central and peripheral pools of apoE contributing to the disease (Narasimhan et al. 2024). Beyond its established role in amyloid deposition and clearance, cholesterol efflux, and tau-mediated neurodegeneration (Husain et al. 2021), peripheral apoE and lipoproteins circulating through the vasculature directly impact cerebrovascular function, therefore affecting brain health. Low-density lipoprotein cholesterol (LDL-C) was highlighted as a causal factor in atherosclerosis (Ference et al. 2017) and, according to the 2024 Lancet report, a midlife risk factor for AD (Livingston et al. 2024). In 559 participants from the ROSMAP followed up for 7 years, LDL-C was associated with all measures of AD neuropathology including neurofibrillary tangles, amyloid, Braak stage, CERAD score, global AD pathology and CAA independent of *APOE* after adjusting for age, sex, cholesterol-lowering medication use, BMI, smoking and education (Aliza P Wingo et al. 2022). Recent National Health and Nutrition Examination Survey (NHANES) analyses linked high LDL to anti-atherogenic high density lipoprotein (HDL) ratios to cognitive decline in men underscoring sex differences in older adults (Wen et al. 2024).

HDL was emphasized as a heterogeneous lipoprotein subclass with diverse vasoprotective and anti-inflammatory functions (W. S. Davidson et al. 2021; W. S. Davidson et al. 2022). However, HDL’s role in AD and cognitive impairment has been debated with some studies showing protective role of HDL cholesterol (HDL-C) and others not finding a difference (Huang et al. 2025), which could be attributed to the heterogeneity, and thereby functionality of HDL particles. Evidence from both genetic and pharmacological in vivo studies support HDL and its major apolipoprotein, apoA-I, decreasing CAA (Lewis et al. 2010; Button et al. 2019; Lefterov et al. 2010; Fernández-de Retana et al. 2017; Zhong et al. 2025) by reducing Aβ binding to collagen-1 (Robert et al. 2020). Moreover, a small fraction (~ 8–10%) of HDL particles contain apoE (Sacks et al. 2022), which may endow HDL with key functional properties. Notably, in mice, liver-expressed apoE4 compromised synaptic plasticity and cognition by impairing cerebrovascular functions while apoE3 plasma from young mice improved cognition and reduced vessel-associated gliosis when transfused into aged mice (Liu et al. 2022), reinforcing the systemic contribution of lipoproteins to AD and VCID. Despite progress, the roles of HDL subfractions and peripheral apoE in modulating cerebrovasculature remain incompletely understood, underscoring the need for better models.

To address this gap, Tetiana Poliakova, a graduate student in Dr. Wellington’s laboratory (University of British Columbia) presented her work on development of a unique mouse model. She aims to reconstitute the expression of cholesteryl ester transfer protein (CETP), a key regulator of LDL-C to HDL-C ratio in humans, in 5xFAD mice to study the effects of peripheral lipids in AD. Because CETP activity increases LDL-C and decreases HDL-C (Poliakova and Wellington 2023), CETP inhibition has been explored in for coronary heart disease with a new promising CETP inhibitor, obicetrapib, currently in phase III clinical trials (Stephen J. Nicholls et al. 2022a). Genetic and pharmacological evidence connects low CETP activity with improved cardiovascular health and longevity (S. J. Nicholls et al. 2022b), and Mendelian Randomization reveals causal associations between CETP and dementia, with Parkinson Disease Dementia (PDD) being particularly robust (Schmidt et al. 2024). Using novel CETPx5xFAD mouse models, Poliakova demonstrated successful “humanization” of the murine lipoprotein profile, enabling translational exploration of CETP’s role in AD and future testing of CETP inhibition in a mouse model. The next steps also include complementary analyses in the Comprehensive Assessment of Neurodegeneration and Dementia: Canadian Cohort Study (COMPASS-ND) to test the hypothesis that CETP activity is elevated in dementia and associated with vascular lesions, cognitive decline, and plasma biomarkers including Aβ42/40, p-tau181, glial fibrillary acidic protein (GFAP) and neurofilament light (NfL), with *APOE* genotype acting as a modifier.

The BROADWAY Phase 3 trial reported that obicetrapib not only reduced LDL-C by 36% and raised HDL-C by 125% in *n* = 1,515 patients with established atherosclerotic cardiovascular disease (ASCVD) but also stabilized plasma AD biomarkers (Michael H. Davidson et al. 2025). In particular, progression of p-tau-217 and the p-tau-217/Aβ42:40 ratio was attenuated, with the most pronounced effects observed in *APOE4* carriers and homozygotes. Parallel improvements were noted across additional AD biomarkers, positioning CETP as a potential therapeutic target for lipid-vascular risk in AD and VCID (Michael H. Davidson et al. 2025).

### Vascular risk score

Expanding on systemic influences, Soumilee Chaudhuri, PhD student in Dr. Andrew Saykin’s laboratory (Indiana University School of Medicine), also presented work integrating genetic epidemiology, single-cell transcriptomics, neuroimaging, and blood biomarkers to interrogate vascular-amyloid interactions. Cardiovascular risk factors were shown to shape cognitive trajectories and plasma biomarker profiles even in amyloid-negative individuals, highlighting vascular health as an independent contributor to preclinical AD. In 526 Korean Brain Aging Study (KBASE) participants, vascular burden significantly lowered baseline cognition in amyloid-negative older adults, while amyloid pathology remained the primary driver of decline in cognitively normal (CN) and mild cognitive impairment (MCI) regardless of the vascular risk status (Chaudhuri et al. 2024).

In the Indiana Memory and Aging Study (IMAS), preliminary biomarker studies revealed stage-specific associations between a vascular risk score (VRS) (hypertension, diabetes, hypercholesterolemia, transient ischemic attack, and stroke) and plasma biomarkers including Aβ42, Aβ40, total tau, NfL, and GFAP. In the CN group, Aβ42/40 and GFAP levels differed significantly between individuals who had high VRS vs. low VRS. Aβ40, Aβ42, and total tau levels differed significantly in the MCI group based on high and low VRS. In the AD group, Aβ40, Aβ42 and NfL biomarker levels differed by VRS groups. In the future, Chaudhuri plans to conduct replication studies and meta-analysis with the ADNI cohort as well as large-scale proteomic screens to further investigate a broad systemic signature of vascular burden, with 134 proteins significantly linked to vascular risk scores in preliminary analyses.

### Hypertension

Dr. Iadecola expanded on the role of hypertension as the major risk factor in both AD and VCID (C. Iadecola and Davisson 2008) and its effect on the neurovasculome. One of the most translationally relevant models of hypertension in rodents is the deoxycorticosterone acetate (DOCA) salt model, which recapitulates salt-sensitive hypertension. It represents around 50% of all cases in humans, is resistant to treatment and associated with brain damage and cognitive impairment (Basting and Lazartigues 2017). DOCA-salt hypertension reduces the CBF flow response to neural activation elicited by mechanical stimulation of the facial whiskers and impairs cognitive function measured by Barnes maze, novel object recognition, and nest-building ability (M. M. Santisteban et al. 2024). Neurovascular and cognitive dysfunction is mediated by interleukin (IL)−17, a cytokine elevated in patients with hypertension. Using reporter mice, the group has shown that DOCA-salt hypertension alters the arachnoid barrier enabling IL-17 secreted by dural T cells to enter the cerebrospinal fluid and perivascular spaces, where it activates IL-17 receptors on BAMs. Consistently, elimination of BAMs, deletion of IL-17 receptor A in brain macrophages, or inhibition of meningeal T-cell activity, restores cognitive function without altering blood pressure, circulating IL-17 levels, or central angiotensin signaling (M. M. Santisteban et al. 2024). In addition to the changes in blood flow and neuroimmune function, Iadecola’s group is now investigating the role of IL-17-mediated tau phosphorylation in cognitive impairment since it was previously shown that dietary salt induces hyperphosphorylation of tau followed by cognitive dysfunction in mice (Faraco et al. 2019).

### New methods to study peripheral influences

Dr. Yang presented his recent work on blood brain barrier (BBB) permeability and its implication for studying peripheral influences on VCID. Using click chemistry, Yang and colleagues tagged and tracked 1000 s of proteins to assess which proteins can cross the BBB. Using mass spectrometry in brain lysates from injected mice, they found 93 proteins from the brain vessels cross the BBB into parenchyma which included carrier proteins complexes like transferrin for iron transport, apolipoprotein A-I (apoA-I) for HDL cholesterol, haptoglobin (Hp) for hemoglobin and vitamin D Binding Protein (DBP) for vitamin D transport. ApoE, however, was only taken up by endothelial cells and was not transported to parenchyma. The outcome was validated using single tagged protein injections in mice to assess cell type specific uptake of the candidate targets including immunoglobulin G (IgG), apoA-I, hemopexin (Hpx), Serpin Family A Member 1 (SERPINA1), alpha-2-HS-glycoprotein (AHSG). It was observed that these proteins were selectively up taken by various cell types: apoA-I and Hpx were abundant in astrocytes, SERPINA1, a protease inhibitor, in microglia and AHSG in both astrocytes and neurons. Identification of BBB crossing proteins and their specific receptors can accelerate drug delivery. ApoA-I uptake in astrocytes was specifically blocked when it was co-injected with an inhibitor for its scavenger receptor SR-B1.

Together, these presentations emphasized the critical influence of peripheral lipid metabolism and cardiovascular risk on brain health. They highlighted the need for improved mechanistic models, multi-omic approaches and integrative cohort studies to define therapeutic entry points for modifying dementia risk through systemic pathways.

## Animal models of VCID

Understanding the underpinnings of VCID remains limited, in part due to a lack of preclinical models that robustly recapitulate vascular pathologies. Multiple talks and a panel at the workshop discussed how to advance VCID models and how to encourage researchers to use better models once they are developed.

Monogenic forms of cerebral small vessel disease (cSVD) including those caused by mutations in *NOTCH3* (cell signaling receptor), *COL4A1* and *COL4A2* (collagen IV, extracellular matrix protein in basement membranes), *TREX1* (3’ DNA exonuclease), *HTRA1* (serine protease), and *FOXC1* (transcription factor) have provided valuable insight into mechanisms underlying vascular dysfunction and neurodegeneration (Rannikmäe et al. 2020). These genetic mice model severe, early-onset phenotypes that are useful in understanding of the mechanisms of vessel wall integrity, basement membrane organization, and endothelial signaling. However, translating the evidence to sporadic, late-onset VCID requires models that better recapitulate human diversity and multifactorial risk. For example, recent efforts include leveraging genetic diversity to improve models of VCID. Examples include B6.Mthfr^677C> T^ mouse used to study SVD associated with reduced activity of methylenetetrahydrofolate reductase (MTHFR), a critical enzyme in the folate and methionine cycles (Reagan et al. 2022); the WSB.*APP/PS1* line on the wild derived mouse background, which exhibits susceptibility to CAA (Onos et al. 2019); and the NZO/HiltJ mouse model of metabolic syndrome linked to vascular dysfunction (MacLean et al. 2023).

Large-scale collaborative platforms such as the Model Organism Development and Evaluation for Late-onset Alzheimer’s Disease (MODEL-AD) Consortium (Oblak et al. 2020) aim to integrate clinical, genetic, and pathological data to generate translationally relevant models of late-onset AD and ADRD which are phenotyped using advanced multi-omics methods to pinpoint vascular and neuroinflammation changes relevant to the changes seen in human in addition to traditionally-studies amyloid beta plaques and neurofibrillary tangles. The late-onset AD 3 series (LOAD3) combines humanized alleles of *APOE*, amyloid precursor protein (APP), and tau (MAPT) to examine how genetic risk and pathology interact (Model-AD).

Using rodents, Dr. Doug Gould, PhD (University of California, San Francisco) models cSVD, which manifests in white matter hyperintensities, subcortical microbleeds, enlarged perivascular spaces and lacunar infarcts and often accompanies VCID. While a minority of cSVD is monogenic, Dr. Gould highlights that advances in understanding monogenic forms of cSVD provide valuable insights that can guide future research into common forms of the disease. Focusing on collagen IV-related disease, Gould underscores the power of forward genetics and modifier screens to uncover gene-environment interactions and allelic heterogeneity. *COL4A1* and *COL4A2* are ancestral members of the collagen superfamily. They form α1α1α2 heterotrimers that are present in every tissue in the animal kingdom (Fidler et al. 2018). Recent studies have illuminated the complexity of *COL4A1* and *COL4A2* mutations, which give rise to highly variable multisystem disorders consisting of varying severity of cerebrovascular, ocular, and muscular phenotypes. Mouse models carrying pathogenic *Col4a1* variants demonstrate that phenotypic severity of ocular anterior segment dysgenesis an intracerebral hemorrhages are modulated by genetic background (Mao et al. 2021). Mechanistic studies in mice revealed that mutations in *COL4A1* disrupt vascular development, leading to small-vessel pathology, recurrent hemorrhagic strokes, and progressive, age-associated macroangiopathy. Disease severity and phenotypic variability were influenced by allelic heterogeneity, genetic background, and environmental modifiers such as vigorous physical activity or anticoagulant use, while pharmacological chaperones such as 4-phenylbutyrate (4PBA) show promise in restoring collagen secretion and mitigating pathology (Jeanne et al. 2015; Kuo et al. 2012). In addition to coding mutation that cause multi-system congenital disorder and are characterized by extracellular collagen deficiency and predominantly hemorrhagic cSVD, Dr. Gould discussed the identification of mutations within the conserved regulatory elements of *COL4A1* that cause pontine autosomal dominant microangiopathy with leukoencephalopathy (PADMAL) family (Verdura et al. 2016). In contrast to coding mutations, these non-coding variants act through post-transcriptional dysregulation, leading to elevated *COL4A1* transcript levels and adult-onset, ischemic phenotypes marked by progressive motor and cognitive impairment. Though rare, these non-coding forms may serve as mechanistic analogues of more common sporadic cSVD. By distinguishing the mechanistic divergence between coding and regulatory mutations, these models are advancing the understanding of how basement membrane biology shapes cerebrovascular integrity and cognitive aging.

### Challenges in modelling VCID

During a panel discussion, Dr. Gareth Howell, PhD (The Jackson Laboratory, and co-organizer of the workshop) emphasised that modeling VCID is challenging since it reflects varied vascular pathologies, and therefore varied mechanisms. Moreover, current preclinical approaches lack age consideration yet cerebrovascular pathologies are most common with aging (Izzo et al. 2018). Further, most animal models focus on one pathology, whereas many cases of VCID include multiple or mixed pathologies. Rodents often used for modelling have relatively less white matter, show limited neurodegeneration, and exhibit a natural resilience to cerebrovascular injury, often necessitating the use of extreme methods to induce pathology. An important question raised by Dr. Howell during the panel concerned the fundamental issue of model interpretation: *What are we truly modeling? Does one pathology drive another*,* or do they evolve independently?* Progress towards improved models will depend on embracing the complexity of VCID, integrating human genetic and environmental risk factors, as well as leveraging genetic diversity of animal models, rather than relying on one genetic context. Approaches like these are being taken in MODEL-AD (Oblak et al. 2020). Ultimately, we need models that reflect the intertwined nature of vascular, metabolic, and neurodegenerative processes to improve translation to the clinic.

## Population and health disparities

Health disparities play a critical role in shaping the risk of VCID. Large cohort studies consistently show that racialized and socioeconomically disadvantaged populations carry a disproportionate burden of hypertension, diabetes, stroke, and small vessel disease core mechanisms driving VCID pathology (Zlokovic et al. 2020; Canavan and O’Donnell 2022).

### Race and ethnicity

In diverse community-based cohorts, Black and Hispanic individuals exhibit higher rates of cognitive impairment and dementia even after adjusting for vascular risk factors and imaging markers, underscoring the compounded influence of social determinants of health, chronic stress, and unequal access to preventive care (Wright et al. 2021). Dr. Adam Brickman shared that racial and ethnic differences in small vessel cerebrovascular disease were evident by midlife. Among Latinx and White adults, brain aging became more pronounced in later life, whereas Black adults exhibited an accelerated trajectory of brain aging already beginning in midlife (Turney et al. 2023).

Dr. Julie Schneider shared that neuropathologic data on cerebrovascular contributors to cognitive decline in older Black adults are limited. In a harmonized autopsy study of 112 Black participants, macroscopic infarcts, microinfarcts, cerebral amyloid angiopathy, and arteriolosclerosis were frequent, with mixed cerebrovascular profiles observed in nearly half of cases (Kapasi et al. 2025). Microinfarcts and basal ganglia arteriolosclerosis were each linked to lower global cognition independent of neurodegenerative pathology, with additional domain-specific associations involving episodic and semantic memory and perceptual speed. Mixed vascular profiles were similarly associated with selective cognitive deficits. In matched analyses comparing Black and White decedents, the burden and cognitive impact of cerebrovascular lesions were comparable across racial groups. These findings highlight the high prevalence and cognitive relevance of vascular brain injury in aging Black adults and underscore the importance of addressing mixed vascular pathology in dementia research (Kapasi et al. 2025).

### Sex differences

Dr. Schneider highlighted that Alzheimer’s dementia often arises from overlapping neuropathologies, yet sex differences in these mixed pathologies are not well characterized. In a combined autopsy cohort of more than 1500 older adults, the frequency of common mixed pathologies was compared across women and men (Barnes et al. 2019). Women were significantly more likely to exhibit AD with concomitant cerebrovascular pathology after adjustment for age at death, education, race, and *APOE4*, whereas men more often showed isolated Lewy body disease. These findings indicate that women have a higher burden of AD with vascular co-pathology, highlighting sex-specific patterns in mixed etiologies of dementia. Together, these disparities highlight an urgent need to integrate vascular biology with social and structural determinants to advance prevention and treatment of VCID across all communities.

## Career development and trainee representation

Held in a residential setting at the Highseas Conference Center at The Jackson Laboratory, the workshop brought together experts in vascular biology, neurodegeneration, ADRD, and computational research to advance understanding of key mechanisms underlying VCID, including small vessel disease, cerebral amyloid angiopathy, and blood–brain barrier dysfunction. The program convened 26 in-person participants spanning multiple career stages (2 faculty, 9 postdoctoral fellows, 11 graduate students, and 4 participants in other roles), with strong gender representation (58% female, based on voluntary self-identification). To promote accessibility and broad participation, 24 attendees (92%) received full or partial scholarships supported by a NIH grant (R13NS141586) and the Diana Davis Foundation. The scientific program featured 12 invited speakers from across the United States as well as Canada and England, providing a rich perspective. Trainees gained hands-on experience in grant conceptualization through structured, study section-style sessions, working directly with accomplished investigators, strengthening both scientific rigor and proposal development skills. The immersive format fostered meaningful faculty–trainee interactions, organic networking, and the formation of new cross-disciplinary collaborations. Post-course evaluations demonstrated strong impact and satisfaction, with the workshop earning a Net Promoter Score of + 50, 100% of respondents agreeing or strongly agreeing that their objectives for attending were met, and over 75% reporting high satisfaction that “the event provided an appropriate balance of scientific overview and specific information” and “overall I was satisfied with the quality of instruction”. Participant feedback also provided constructive guidance, identifying opportunities to further enhance future iterations of the workshop by optimizing the grant-writing training component. Overall, the workshop successfully trained a diverse cohort of emerging investigators, catalysed new collaborative partnerships, and empowered future leaders poised to advance therapeutic discovery across the VCID and ADRD spectrum. Based on the success of the first VCID workshop, The Jackson Laboratory will host the second workshop, to be held on April 28-May 1, 2026 at Washington University in St Louis (Vascular Contributions to Cognitive Impairment and Dementia workshop).

## Conclusions and future directions

Overall, the first VCID Workshop was a great success, exceeding expectations for content, interdisciplinary training, and collaboration objectives. The organizers recognize the need to continually evaluate the scope and content to ensure future workshops remain current. Vascular health is a central and often underappreciated determinant of cognitive trajectories in aging and disease. The 2025 VCID Workshop highlighted a key message: cerebrovascular dysfunction is not just a cofactor but a key driver of AD and related dementias. Neurovascular dysfunction and vascular injury do not simply indicate the loss of cerebral blood flow and brain oxygenation, but influence immune, clearance, and trophic dysfunctions. SVD, CAA, BBB breakdown, and impaired CBF interact to create a vulnerable neural environment to accelerate neurodegeneration, exacerbate proteinopathies, and diminish cognitive resilience.

Despite the traditional categorical view of dementia subtypes, most dementia cases exhibit mixed pathologies with vascular injury being particularly common. Sex, race, and *APOE* genotype further shape the burden and impact of vascular injury, highlighting the need to integrate demographic and biological context into both research and clinical practice. Studies presented at this workshop underscores how cSVD including CAA, white matter vulnerability, vascular interactions of tau, and glial activation collectively contribute to the consequences of age related and traumatic cerebrovascular injury. Beyond intrinsic cerebrovascular changes, peripheral physiology plays an important role in VCID development with cardiovascular risk factors and inflammatory pathways shaping brain vulnerability before clinical symptoms. The workshop also emphasized advances in genomics, spatial transcriptomics, blood-based biomarker profiling, and high-resolution imaging which are all transforming our ability to map vascular and neurodegenerative processes with exceptional precision. These technologies will help us to investigate the molecular signatures of cerebrovascular aging, clarify causal mechanisms, and identify therapeutic targets.

Looking ahead, the field must prioritize longitudinal and mechanistically anchored research to fully define how vascular dysfunction interacts with neurodegeneration across the lifespan. One of the priorities remains to develop translationally relevant animal models to study VCID. These unmet needs were part of the motivation for the creation of the VCID Centers without Walls (CWOW), an NINDS-funded collaboration between six centers distributed across the US. Conceptualized by Dr. Roderick Corriveau and NINDS staff, VCID CWOW are aiming to use state-of-the-art approaches, including multi-omics, to identify and prioritize novel therapeutics approaches to treat VCID. Ultimately, treating the cerebrovasculature may represent one of the most impactful strategies for preventing or mitigating dementia, offering new hope for modifying disease course in both Alzheimer’s disease and its related disorders.

## Key Messages

- Vascular pathology is highly prevalent in aging, and is a major underrecognized contributor to cognitive decline and dementia, and so the progress in understanding and treating vascular contributions to cognitive impairment remains limited.
- Dementia is increasingly understood as a mixed etiology disorder, where cerebral small vessel disease, cerebral amyloid angiopathy, blood brain barrier dysfunction, and neurodegenerative pathology frequently coexist and jointly drive cognitive decline.
- Advances in omics and biomarker technologies are enabling deeper mechanistic insight into how aging, vascular health, inflammation, and neurodegeneration interact to cause Alzheimer’s disease and dementia.•Systemic metabolic and cardiovascular factors directly shape cerebrovascular resilience, with dyslipidemia, hypertension, diabetes, and systemic inflammation accelerating vascular injury and cognitive decline even in amyloid-negative individuals.
- Peripheral lipoproteins and apolipoproteins exert direct effects on the cerebrovasculature, influencing endothelial function, vascular inflammation, and amyloid handling; emerging models now enable mechanistic dissection of these pathways.
- BBB permeability determines how circulating proteins, metabolites, and inflammatory signals access the brain, and recent proteomic tracking studies reveal selective uptake of peripheral proteins by distinct CNS cell types, illustrating direct systemic-to-brain communication routes relevant to VCID.
- Systemic vascular risk scores correlate with shifts in plasma AD biomarkers, suggesting that peripheral risk burden influences both peripheral and central signatures of neurodegeneration.
- Loss of coordinated neurovascular unit activity reduces the brain’s ability to meet metabolic demand, contributing to cognitive impairment through chronic hypoxia, white matter degeneration, and reduced synaptic plasticity.
- White matter hyperintensities burden increased risk of AD and dementia, reflecting the intrinsic vascular vulnerability of white matter, which relies on small, distal arterioles highly susceptible to compromise.
- TBI disrupts the blood brain barrier and cerebral blood flow, triggers white matter associated glial activation, and drives mural cell dysfunction that impairs tau clearance and amplifies inflammation, collectively linking vascular injury to chronic neurodegeneration.
- Animal models of monogenic forms of cerebral small vessel disease provide mechanistic insights into vascular dysfunction but translating the evidence to sporadic late-onset VCID requires models that better recapitulate human diversity and multifactorial risk.
- Vascular pathology and associated dementia vary by sex and race, with women and Black adults bearing disproportionate mixed vascular burdens, underscoring the need to account for demographic disparities in dementia mechanisms and risk.

## Data Availability

No datasets were generated or analysed during the current study.
